# Association between plasma trimethylamine N-oxide and coronary heart disease: new insights on sex and age differences

**DOI:** 10.3389/fcvm.2024.1397023

**Published:** 2024-10-07

**Authors:** Yangyang Sun, Xipeng Lin, Zhihao Liu, Lihua Hu, Pengfei Sun, Geng Shen, Fangfang Fan, Yan Zhang, Jianping Li

**Affiliations:** ^1^ Department of Cardiology, Peking University First Hospital, Beijing, China; ^2^ Department of Cardiology, Tianjin Medical University General Hospital, Tianjin, China; ^3^ Key Laboratory of Molecular Cardiovascular Science of Ministry of Education, Peking University, Beijing, China

**Keywords:** trimethylamine N-oxide, coronary heart disease, sex differences, age differences, gut microbiota

## Abstract

**Aim:**

Elevated plasma trimethylamine N-oxide (TMAO) is related to atherosclerosis. Whether the relationship of TMAO and coronary heart disease (CHD) is influenced by sex or age is uncertain. We aim to explore the sex and age differences in the relationship between plasma TMAO and CHD risk and severity.

**Methods:**

A case–control study was conducted in patients undergoing elective coronary angiography. Matched by sex, age (±2 years), and operation date (±180 days), a total of 429 CHD case–control pairs were included. Plasma TMAO was quantified using liquid chromatography–tandem mass spectrometry. Logistic regression analyses were performed to evaluate the association between plasma TMAO and CHD risk and severity.

**Results:**

The overall median (interquartile range) plasma TMAO level was 0.11 (0.06–0.18) μg/ml. After stratification by sex and age, and adjustment for common CHD risk factors, the association between TMAO and CHD risk was significant in the older (≥65 years) male subgroup [odds ratios (OR) = 1.57, 95% confidence interval (CI): 1.09–2.28, *P* = 0.016], but not in other sex–age subgroups (all *P* > 0.05). The relationship of plasma TMAO and CHD risk was modified by age (adjusted *P*_interaction_ = 0.001) in male individuals. Plasma TMAO was also associated with a higher risk of multi-vessel disease in male patients with CHD (OR = 1.65, 95% CI: 1.18–2.32, *P* = 0.004), but not in females.

**Conclusions:**

Plasma TMAO is significantly positively associated with the risk and severity of CHD in Chinese men. Age has an interactive effect on the relationship between plasma TMAO and CHD risk in men. Our findings warrant further investigation.

## Introduction

Cardiovascular diseases (CVD) remain the major leading cause of mortality and rising healthcare costs worldwide (1, 2). In the Global Burden of Disease Study 2019, prevalent cases of CVD increased from 271 million in 1990 to 523 million in 2019, and deaths of CVD increased from 12.1 to 18.6 million over 30 years (2), which created a great burden on the global economy and health. To better deal with this public issue, a search for biological markers for CVD risk prediction has been ongoing. Trimethylamine N-oxide (TMAO) has emerged as an important metabolite of gut microbiota and has attracted great attention. In 2011, TMAO was first identified as a biomarker associated with atherosclerosis in a large clinical cohort study and in animal experiments (3), indicating a causal relationship between high TMAO levels and CVD risk. Since then, numerous studies have confirmed that elevated TMAO is related to an increased risk of CVD, stroke (4), aortic aneurysm (5), all-cause mortality (6), and major adverse cardiac events (MACE) (7, 8). With increasing supporting evidence, the atherogenic effect of TMAO has been accepted.

TMAO is a product of gut microbiota, inseparable from dynamic interactions between diet and host (9). Its formation occurs via a two-step meta-organismal pathway in mammals. The first step is the synthesis of trimethylamine (TMA) in the host gut. Diets rich in choline, betaine, L-carnitine, and phosphatidylcholine are common TMA precursors (3, 10). They can be converted to TMA by gut microbiota after intake. Then, TMA is transported via the portal circulation to the liver and oxidized to TMAO mainly by the hepatic enzymes flavin monooxygenase 3 (FMO3) (3). The majority of TMAO is eliminated unchanged by the kidneys within 24 h. Renal dysfunction leads to an accumulation of TMAO in the body (11). TMAO was initially treated as a metabolic waste. Nowadays, increasing evidence shows that TMAO actively participates in various biological reactions, affecting the activities of hormones and enzymes within a human body.

Plasma TMAO is a biomarker affected by dietary patterns and of regional characteristic. Many published studies have revealed the prediction value of plasma TMAO levels for CVD risk, but studies in China are lacking. The atherogenic effect of TMAO is well-known. Meta-analysis indicated a significant relation between elevated plasma TMAO levels and increased incidence of MACE in patients with CVD (12). Whether this association varies in different sex and age populations is unknown. Compared to young people, the elderly tend to have higher plasma TMAO levels (13). FMO3 expression is regulated by sex hormones, with FMO3 activity higher in females than males (14). Therefore, it is proposed that the association between plasma TMAO and CVD differs among patients depending on age and gender. Few studies have explored the possibility. In this study, we focus on age (younger or older than 65 years) and sex differences, aiming to explore the association between plasma TMAO levels and coronary heart disease (CHD) risk and severity in patients undergoing diagnostic coronary angiography in China.

## Materials and methods

### Study population and design

The study enrolled participants at the Department of Cardiology, Peking University First Hospital, between January 2016 and December 2019. We adopted a retrospective case–control study design. Adults (aged ≥ 18 years) with symptoms or signs of CHD who underwent elective coronary angiography met the inclusion criteria. Exclusion criteria included the following: a history of diagnosed CHD by physician; serum cardiac troponin I (cTnI) ≥0.04 ng/ml; refusal to supply a venous blood sample; and inability to provide informed consent. Information on related medical history, physical examination, and blood tests was obtained from the electronic medical record system. Blood samples were collected prior to cardiac catheterization procedure, processed soon after collection, and frozen at −80℃ until testing could be done. The study was conducted in accordance with the Declaration of Helsinki and was approved by the ethics review board of Peking University First Hospital (Beijing, China). Written informed consent was provided by all participants.

**Figure 1:**
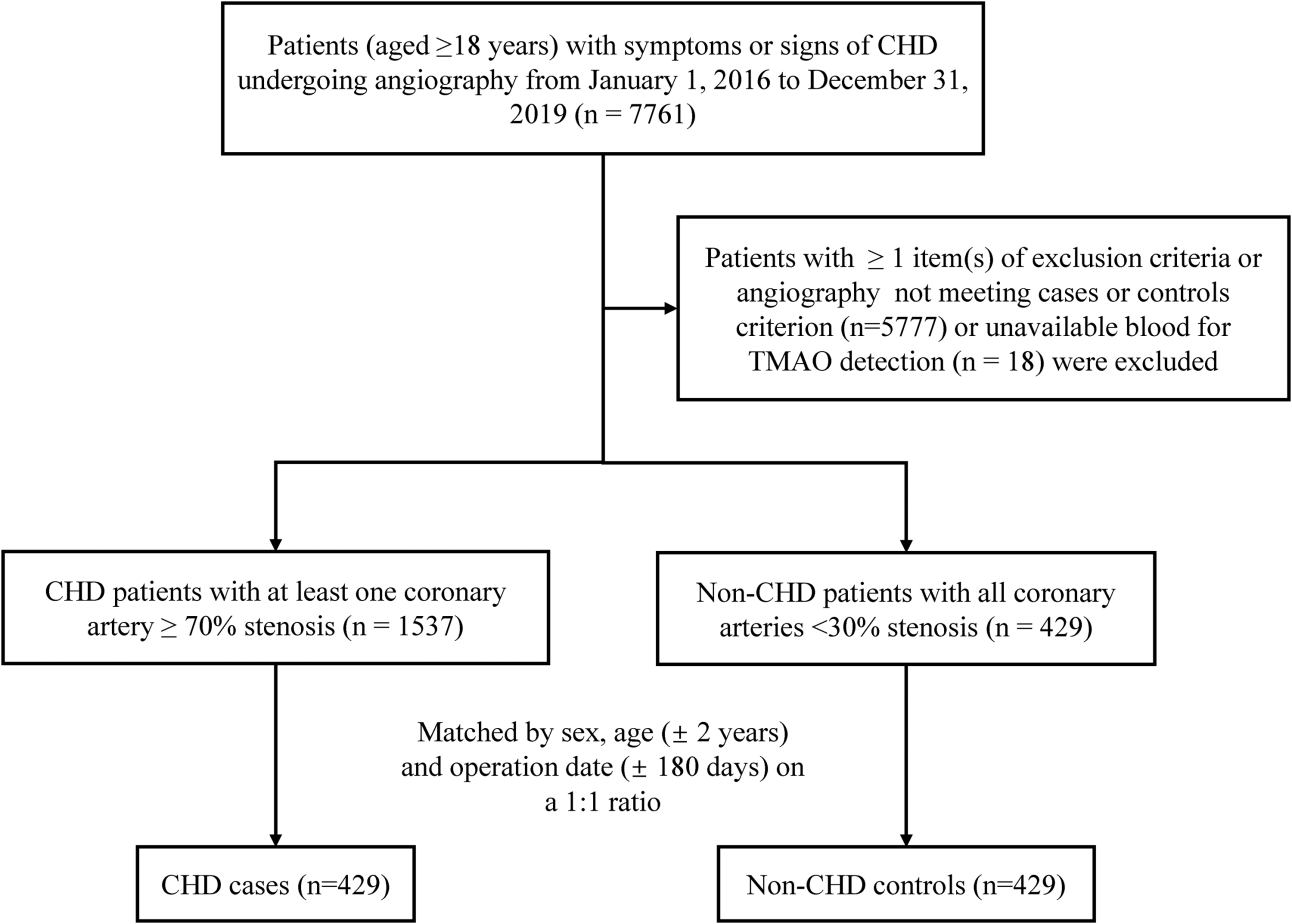
Study flow chart. CHD, coronary heart disease; TMAO, trimethylamine N-oxide.

A flowchart of the study is shown in Figure 1. A total of 7,761 patients undergoing angiography were initially screened. We established a case–control cohort based on this population. To appropriately amplify the differences in disease severity between the two subgroups, cases were defined as having at least one major coronary artery (including left main trunk, left anterior descending, left circumflex, and right coronary artery) ≥70% stenosis diagnosed by angiography. Controls were selected from those non-CHD individuals assessed by cardiac catheterization who had <30% coronary stenosis. A total of 1,537 incident CHD cases and 429 non-CHD controls were finally identified. Matched by sex, age (±2 years), and operation date (±180 days) on a 1:1 ratio, the final cohort for analysis included 429 matched pairs of CHD cases and controls.

### Laboratory testing of plasma TMAO

Plasma TMAO concentration was quantified using liquid chromatography–tandem mass spectrometry (15). The linear measurement range for plasma TMAO was 0.02–1.28 μg/ml. The average extraction recovery rate was above 90%. Both within and between batch precision of duplicate samples was <15%.

### Assessment of coronary atherosclerotic severity

A diseased vessel was defined as any of the major coronary arteries diagnosed with ≥50% stenosis under angiography. To assess the severity of coronary atherosclerosis, patients with CHD in the case group were divided based on the number of diseased vessels present, into the single-vessel disease (SVD, *n* = 128) group and the multiple-vessel disease (MVD, *n* = 301) group, in which patients had at least two diseased vessels.

### Covariates and stratified analysis

In this study, information including age, sex, body mass index (BMI), blood pressure, operation date, comorbidities, medication use, cigarette and alcohol consumption, family history, hemoglobin, fasting plasma glucose concentration, plasma homocysteine (Hcy) concentration, plasma high-density lipoprotein cholesterol (HDL-C) concentration, plasma low-density lipoprotein cholesterol (LDL-C) concentration, plasma creatinine concentration, and D-Dimer concentration were collected from medical records. Smoking or drinking status was divided into three categories (never, ever, currently) according to related history. Physician-diagnosed hypertension, hyperlipidemia, and diabetes were confirmed by the disease diagnostic criteria, according to medical history and biomarkers. Given the potential correlations between renal function and TMAO, creatinine measured by enzymatic assay was also collected as a covariate. Thus, variables used in the fully adjusted model included the continuous variables including age (years), BMI (kg/m^2^), Hcy (μmol/L), and creatinine (μmol/L); and the categorical variables included sex (male/female), hypertension (yes/no), diabetes (yes/no), hyperlipidemia (yes/no), smoking status (never, ever, currently), drinking status (never, ever, currently), and family history of CHD (yes/no).

We intended to examine whether sex and age affected the association between plasma TMAO and CHD. The whole population was first stratified by sex, which was an essential and natural factor. Then, the male and female subgroups were further stratified by age, using 65 years as the dividing point. This age was chosen because it is advocated as the boundary for dividing middle-aged and elderly people in China. In the 2019 Chinese Society of Cardiology (CSC) guidelines for the diagnosis and management of patients with ST-segment elevation myocardial infarction (16), being ≥65 years is listed as a risk factor for CHD. Many published studies also used a cut-off of 65 for age subgroup analysis. Therefore, the study population was stratified by age of 65 years after stratification by sex.

### Statistical analysis

Continuous variables were described as mean ± standard deviation (SD) for normally distributed variables or as median (interquartile range) for skewed distributed variables. Categorical variables were presented as number (%). We used Student's *t*-tests or non-parametric tests for continuous variables and *χ*^2^ tests for categorical variables to examine the differences between two subgroups. Because the distribution of TMAO values was skewed toward the upper end, TMAO values were log_2_-transformed to improve normality of the distribution. When verifying the relationship between plasma TMAO and CHD, TMAO values served as a continuous variable with per 1 μg/ml increment in log_2_ (TMAO) or as a tertile categorical variable, with tertile 1 as the reference group. Logistic regression models were performed to estimate odds ratios (OR) and confidence intervals (CI). *P* for trend was acquired by transforming the median value of each tertile as a continuous variable. Stratified and interactive analyses were examined according to different sex and age by likelihood ratio tests. The smooth curve was drawn by a cubic spine function model to present the shape of the dose–response relation of plasma TMAO and MVD risk. A two-sided *P*-value <0.05 was considered of statistical significance. The analyses were performed using R software version 4.0.2.

## Results

### Baseline characteristics

The study totally involved 429 CHD cases and 429 matched controls for analysis. The baseline characteristics of participants in the different sex and age groups are presented in Table 1. Overall, the mean ± SD age was 63.5 ± 10.4 years; 53.1% were women; 69.9%, 42.1%, and 78.0% subjects had a discharge diagnosis of hypertension, diabetes, and hyperlipidemia, respectively. The median (interquartile range) plasma TMAO concentration was 0.11 (0.06–0.18) μg/ml. There were statistically significant differences in BMI, blood pressure, smoking status, hyperlipidemia diagnosis, antihypertensive and glucose-lowering drugs use, hemoglobin, HDL-C, LDL-C, creatinine, and D-Dimer levels (all *P* < 0.05) between older (≥65 years) and younger (<65 years) groups in men, not including plasma TMAO (*P* = 0.562). In women, the differences between the two age subgroups mainly existed in hypertension, CHD family history, antihypertensive drugs use, hemoglobin, creatinine, D-Dimer, and TMAO levels (all *P* < 0.05). The cases accounted for 53.7% and 54.2%, respectively, in the men and women of older groups.

**Table 1:** Characteristics of participants in different sex and age subgroups.

Characteristic^a^	Male			Female		
	<65 years	≥65 years	*P-*value	<65 years	≥65 years	*P-*value
*N*	255	147		203	253	
BMI (kg/m^2^)	26.7 ± 3.2	25.3 ± 3.5	<0.001	26.2 ± 4.1	25.9 ± 3.8	0.440
SBP (mmHg)	130.9 ± 15.6	134.4 ± 16.7	0.040	131.9 ± 17.1	134.7 ± 16.8	0.078
DBP (mmHg)	79.8 ± 11.1	73.6 ± 10.9	<0.001	75.2 ± 9.9	70.3 ± 9.9	<0.001
Smoking status (%)			<0.001			0.780
Never	48 (19.6)	46 (32.2)		181 (91.9)	215 (90.0)	
Ever	79 (32.2)	62 (43.4)		5 (2.5)	7 (2.9)	
Current	118 (48.2)	35 (24.5)		11 (5.6)	17 (7.1)	
Drinking status (%)			0.059			0.789
Never	96 (38.7)	67 (48.2)		190 (95.0)	234 (96.3)	
Ever	47 (19.0)	30 (21.6)		3 (1.5)	3 (1.2)	
Current	105 (42.3)	42 (30.2)		7 (3.5)	6 (2.5)	
Comorbidities, *N* (%)						
Hypertension	161 (63.1)	106 (72.1)	0.067	138 (68.0)	195 (77.1)	0.030
Diabetes	168 (65.9)	85 (57.8)	0.107	90 (44.3)	122 (48.2)	0.408
Hyperlipidemia	209 (82.0)	106 (72.1)	0.021	155 (76.4)	199 (78.7)	0.558
Family history of CHD	92 (38.2)	43 (31.6)	0.202	91 (47.6)	68 (29.8)	<0.001
Medication use, *N* (%)						
Antihypertensive	121 (47.5)	85 (57.8)	0.045	105 (51.7)	165 (65.2)	0.004
Glucose-lowering	57 (22.4)	47 (32.0)	0.034	69 (34.0)	90 (35.6)	0.724
Lipid-lowering	112 (43.9)	66 (44.9)	0.849	94 (46.3)	139 (54.9)	0.067
Laboratory biomarkers						
Hemoglobin (g/L)	146.7 ± 12.3	138.7 ± 13.3	<0.001	129.6 ± 12.4	126.8 ± 12.0	0.016
Glucose (mmol/L)	6.5 ± 2.2	6.9 ± 2.9	0.145	7.3 ± 3.3	7.3 ± 3.5	0.952
HDL-C (mmol/L)	1.0 ± 0.2	1.0 ± 0.2	0.035	1.1 ± 0.2	1.1 ± 0.3	0.981
LDL-C (mmol/L)	2.4 ± 0.8	2.1 ± 0.6	<0.001	2.4 ± 0.8	2.3 ± 0.8	0.239
Hcy (μmol/L)	14.4 (11.1–18.9)	15.2 (11.5–19.1)	0.745	11.5 (9.1–15.4)	12.9 (10.0–15.6)	0.106
Creatinine (μmol/L)	81.7 (74.6–90.9)	88.8 (79.9–99.2)	<0.001	63.6 (57.5–71.2)	69.5 (61.9–78.4)	<0.001
D-Dimer (mg/L)	0.06 (0.04–0.09)	0.10 (0.07–0.16)	<0.001	0.08 (0.05–0.13)	0.11 (0.07–0.19)	<0.001
Cases, *N* (%)	122 (47.8)	79 (53.7)	0.255	91 (44.8)	137 (54.2)	0.048
TMAO (μg/ml)	0.10 (0.06–0.17)	0.11 (0.06–0.17)	0.562	0.09 (0.06–0.15)	0.12 (0.08–0.22)	<0.001

CHD, coronary heart disease; BMI, body mass index; SBP, systolic blood pressure; DBP, diastolic blood pressure; LDL-C, low-density lipoprotein cholesterol; HDL-C, high-density lipoprotein cholesterol; Hcy, homocysteine; TMAO, trimethylamine N-oxide;.

^a^ Data are presented as number (%) or mean ± standard deviation or median (interquartile range) depending on the distribution.

In addition, baseline characteristics of cases and controls divided by sex are presented in Supplementary Table 2. In both men and women, the CHD cases were more likely to be smokers, to have a diagnosis of diabetes, and to use more glucose-lowering and lipid-lowering drugs than controls (all *P* < 0.05). In women, CHD cases also had higher rates of hypertension and hyperlipidemia, higher plasma creatinine levels, and lower hemoglobin and plasma HDL-C levels than the controls (all *P* < 0.05).

### Association between plasma TMAO and CHD risk in different sex and age subgroups

The associations between plasma TMAO and CHD risk in the different sex and age subgroups are presented in Table 2. As presented in the crude model, the association between plasma TMAO and CHD risk was significant in the older (≥65 years old) male subgroup (OR = 1.47, 95% CI: 1.09–1.97, *P* = 0.012), but not in the younger (<65 years old) male subgroup, older female subgroup, and younger female subgroup (all *P* > 0.05). After adjustments for common CHD risk factors (BMI, smoking status, drinking status, hypertension diagnosis, diabetes diagnosis, hyperlipidemia diagnosis, family history of CHD, creatinine, and Hcy), elevated TMAO was still significantly associated with higher CHD risk in the older male subgroup (OR = 1.57, 95% CI: 1.09–2.28, *P* = 0.016), but not in any other subgroup (all *P* > 0.05). Age had an interactive effect on the relationship between plasma TMAO and CHD risk in male patients (adjusted *P*_interaction_ = 0.001). TMAO values as a tertile categorical variable for analysis are provided in Supplementary Table 3.

**Table 2:** Association between plasma TMAO and risk of CHD in patients stratified by sex and age.

TMAO^a^ (μg/ml)	Cases/controls	Crude model		*P* for interaction	Adjusted model		*P* for interaction
		OR (95% CI)	*P*-value		OR (95% CI)	*P*-value	
Male							
<65 years	122/133	0.84 (0.69–1.02)	0.083	0.002	0.87 (0.70–1.09)	0.242	0.001
≥65 years	79/68	1.47 (1.09–1.97)	0.012		1.57 (1.09–2.28)	0.016	
Total	201/201	1.00 (0.86–1.17)	0.964		1.04 (0.87–1.24)	0.681	
Female							
<65 years	91/112	1.16 (0.93–1.44)	0.196	0.322	1.10 (0.84–1.45)	0.478	0.862
≥65 years	137/116	0.99 (0.80–1.22)	0.936		1.09 (0.84–1.42)	0.516	
Total	228/228	1.09 (0.94–1.27)	0.239		1.06 (0.89–1.27)	0.520	

CHD, coronary heart disease; TMAO, trimethylamine N-oxide; BMI, body mass index; Hcy, homocysteine.

The adjusted model was adjusted for BMI, smoking status, drinking status, hypertension diagnosis, diabetes diagnosis, hyperlipidemia diagnosis, family history of CHD, creatinine, and Hcy.

^a^ TMAO value was log_2_-transformed.

### Association between plasma TMAO and numbers of diseased vessels in patients with CHD

**Figure 2:**
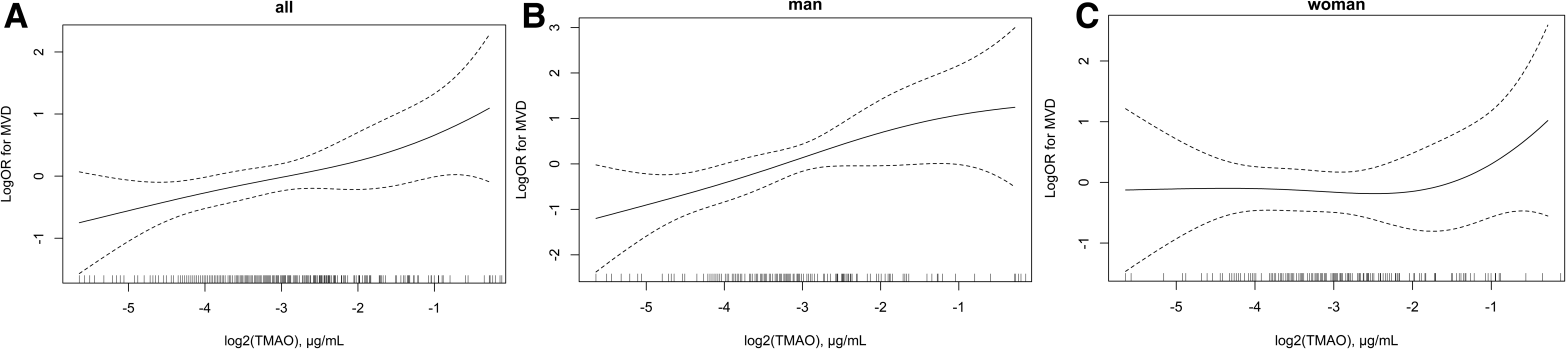
Association between plasma log_2_ (TMAO) and risk of multi-vascular diseases in patients with CHD. **(A)** Overall population, **(B)** men, and **(C)** women. Adjustment factors included sex (only for overall population), age, BMI, smoking status, drinking status, hypertension diagnosis, diabetes diagnosis, hyperlipidemia diagnosis, family history of CHD, creatinine, and Hcy. CHD, coronary heart disease; TMAO, trimethylamine N-oxide; MVD, multiple-vessel disease; SVD, single-vessel disease; BMI, body mass index; Hcy, homocysteine.

The association between plasma TMAO and severity of atherosclerosis in patients with CHD stratified by sex is presented in Table 3. Lesion severity was mainly assessed by using the number of diseased vessels, with patients grouped into SVD and MVD subgroups as defined previously. Overall, each 1 μg/ml increment in log_2_ (TMAO) was associated with a 35% higher risk of MVD in the CHD population in the adjusted model (OR = 1.35, 95% CI: 1.09–1.66, *P* = 0.005). The association was statistically significant in male patients with CHD after adjusting for common CHD risk factors (OR = 1.65, 95% CI: 1.18–2.32, *P* = 0.004), but not in females (OR = 1.13, 95% CI: 0.84–1.51, *P* = 0.425). Using tertile 1 of TMAO as reference, among male patients with CHD, being in the highest TMAO tertile was also statistically associated with MVD in the adjusted model (OR = 3.54, 95% CI: 1.39–8.99, *P* = 0.008). Due to the small sample in some subgroups, age stratification was not further performed. The association between plasma TMAO and risk of MVD in patients with CHD is further presented as smooth curves in Figure 2, which also show the sex differences clearly. Scatter plots that included individual data are shown in the Supplementary Material.

**Table 3:** Association between plasma TMAO and multi-vascular diseases in patients with CHD stratified by sex.

TMAO (μg/ml)	MVD/SVD	Crude model		Adjusted model	
		OR (95% CI)	*P*-value	OR (95% CI)	*P*-value
Overall					
Continuous^a^	301/128	1.34 (1.12–1.60)	0.001	1.35 (1.09–1.66)	0.005
Tertiles					
T1 (≤0.080)	90/52	Ref.		Ref.	
T2 (0.080–0.152)	99/44	1.18 (0.72–1.93)	0.504	1.07 (0.61–1.88)	0.820
T3 (>0.152)	112/32	2.13 (1.26–3.62)	0.005	2.14 (1.15–3.95)	0.016
*P* for trend		0.005		0.018	
Male					
Continuous^a^	145/56	1.48 (1.12, 1.95)	0.006	1.65 (1.18, 2.32)	0.004
Tertiles					
T1 (≤0.075)	42/24	Ref.		Ref.	
T2 (0.075–0.139)	48/19	1.44 (0.70–3.00)	0.325	1.75 (0.73–4.19)	0.209
T3 (>0.139)	55/13	2.42 (1.10–5.30)	0.028	3.54 (1.39–8.99)	0.008
*P* for trend		0.027		0.008	
Female					
Continuous^a^	156/72	1.26 (1.00–1.60)	0.051	1.13 (0.84–1.51)	0.425
Tertiles					
T1 (≤0.083)	48/28	Ref.		Ref.	
T2 (0.083–0.159)	51/25	1.19 (0.61–2.32)	0.610	0.75 (0.33–1.70)	0.489
T3 (>0.159)	57/19	1.75 (0.87–3.52)	0.116	1.07 (0.44–2.62)	0.887
*P* for trend		0.118		0.933	

CHD, coronary heart disease; TMAO, trimethylamine N-oxide; MVD, multiple-vessel disease; SVD, single-vessel disease; BMI, body mass index; Hcy, homocysteine.

The adjusted model was adjusted for sex (only for overall population), age, BMI, smoking status, drinking status, hypertension diagnosis, diabetes diagnosis, hyperlipidemia diagnosis, family history of CHD, creatinine, and Hcy.

^a^ TMAO value was log_2_-transformed.

## Discussion

As far as we known, this study was the first to examine the relationship between plasma TMAO levels and CHD risk in different sex and age populations. The main results included the following: (1) higher plasma TMAO concentration was associated with a higher risk of CHD in elderly male patients, independent of most traditional CHD risk predictors; (2) there existed a significant interactive effect of age and TMAO concentration on CHD risk in male patients; (3) higher plasma TMAO level was associated with a higher risk of MVD presence overall, and especially in male patients with CHD.

With the proposal of gut microbiota metabolism affecting progression of CVDs, TMAO has been widely studied and its atherogenic roles were confirmed in various disease states. For stable patients with CHD undergoing cardiac assessment, elevated plasma TMAO was identified to be related to a higher risk for CVD (3) and MACE (7, 8), and portended a higher long-term mortality risk regardless of optimal medical treatment (6). Among patients suspected or diagnosed of acute coronary syndrome, plasma TMAO was also independently associated with risk of short-term MACE and long-term mortality (17, 18). A meta-analysis containing 10,301 individuals showed that elevated plasma TMAO could increase risk of MACEs by 58% in patients with CHD (*P* < 0.001) (12). The association between plasma TMAO concentration and incidence of MACE presented as a “J” shape by the dose–response analysis (*P* = 0.033), which indicated an association of HR >1 when TMAO was over 5.1 μmol/L. In our study, we also found a positive correlation between plasma TMAO and risk of CHD, although not in the whole population. The dose-dependence suggests that TMAO must reach a certain level to have adverse effects. Its effect on atherosclerosis might be interfered by other factors.

In addition, higher levels of circulating TMAO were correlated with more severe CHD lesions. Yu et al. (11) reported that higher plasma TMAO was significantly associated with triple vessel diseases and SYNTAX score >22 in diabetic patients with estimated glomerular filtration rate <60 ml/min/1.73 m^2^. Senthong et al. (19) showed that elevated plasma TMAO was independently related to a higher SYNTAX score and could predict the presence of diffuse lesions. Other studies also identified a significant association between higher TMAO and higher Gensini score (20), heavier vascular calcification (21), and increased incidence of vulnerable plaques (characteristic of thin-cap fibroatheroma) (22). Existing evidence suggests that plasma TMAO plays a key role in the development and progress of atherosclerosis. Most published studies focused on the whole population, paying little attention to the possible impact of sex or age differences on the TMAO–CHD association. But this impact from sex and age differences does exist.

Several studies have reported the association between TMAO and CHD risk in different sex subgroups. In 2013, Tang et al. (7) discovered the prognostic value of elevated plasma TMAO levels for cardiovascular risk was reduced in younger (<65 years old) and women subgroups, but not providing possible reasons. In a clinical cohort of 2,235 stable patients with CHD, the prognostic value of elevated plasma TMAO for 5-year mortality was reduced in the female compared to the male subgroup (6). In a small cross-sectional study, plasma TMAO was confirmed as an independent risk factor of CHD and severe artery stenosis. It was more sensitive in men than in women based on receiver operating characteristic analysis (23). Further, Fu et al. (24) enrolled 1,032 peritoneal dialysis patients in their prospective study. Over a median follow-up of 63.7 months, they observed elevated plasma TMAO was independently associated with increased risk of both all-cause (*P* = 0.013) and cardiovascular mortality (*P* = 0.038) in men but not in women. These results all exhibited a sex discrepancy. Accordingly, our study divided the whole population into four subgroups by sex and age, and showed that TMAO was statistically significant to predict risk and severity of CHD in male individuals, but not females. The result is consistent with previous findings and intriguingly identifies age as an interactive factor in the predictive value of TMAO. Possible reasons are discussed in the following.

Based on the metabolic process of TMAO, several factors could affect plasma TMAO levels and its prognostic value for CHD. First is dietary habits and gut microbiota composition. Men were reported to consume more red meat and fat and fewer fiber-rich foods than women (13, 25, 26), which might result in accumulation of TMA-containing compounds, increasing TMAO levels and CVD risks. Gut microbiota composition is influenced by sex, diets, medication use, and illness. Women harbor a higher ratio of Firmicutes/Bacteroidetes in the gut compared to men (23, 27). Gut bacteria in the Firmicutes phylum can produce short-chain fatty acids, which were elucidated to have cardiovascular protective effects (28), and might alleviate the atherogenic effects of other gut microbiota metabolites like TMAO. Second is the sexual dimorphism of hepatic FMO3. FMO3 is crucial in the synthesis pathway of TMAO. It is reduced in males compared to females in both humans and mice. Animal studies indicated that the reduced FMO3 expression was primarily attributed to downregulation of androgens, and upregulation of estrogen (14). Therefore, the difference is genetically determined and might modify the association between TMAO and CHD to some degree. Of note, Bennett et al. (14) also discovered a sex difference in fractional excretion of TMAO in mice, with an obvious increase in males compared to females when fed the same control diets. The phenomenon needs further verification in humans. In summary, both the published literature and our study support the sex differences in the relationship between plasma TMAO levels and CHD risk or severity. That is, the relationship was more pronounced in males compared to females, which could be explained by dietary habits, microbiota background, and metabolic and genetic differences. In our study, CHD risk factors in men and women also differed. Men were more likely to be younger, to have lower plasma LDL-C levels and higher plasma creatinine and Hcy values, to be smokers and drinkers, and to have lower rates of hypertension, diabetes, and medication use than women (data not shown here). Hence, the complex interactions between TMAO and CHD risk factors should be considered in studying the role of sex.

Plasma TMAO levels increase with age. There are several possible mechanisms. First, more than 90% of TMAO is excreted through the kidneys (14). Circulating TMAO levels increase with the decline of renal function (11). Prone to various chronic diseases, the elder usually have poorer renal function than the younger people, and thus tend to have higher TMAO levels correspondingly. In addition, epithelial integrity of the colon might reduce and intestinal permeability may increase with age. This promotes the influx of bacterial metabolites, including TMA and TMAO (13, 29). In a German cohort including 425 participants, Rath et al. (13) constructed a structural equation model that indicated higher plasma TMAO with increasing age were mediated by diet and TMA-forming bacteria. They also reported that TMAO was significantly associated with intima-media thickness only in patients >65 years of age (*P* = 0.018), which was consistent with our discovery. In our study, we found higher plasma TMAO was associated with higher CHD risk in older male patients, independent of most traditional CHD risk predictors. Age had an interactive effect on the association between plasma TMAO concentration and CHD risk in males. This age effect could be attributable to the changing host physiology and loss of organ function, diverse diets, and gut bacteria composition, partly the same as sex differences.

TMAO has the potential to accelerate atherosclerosis progress, leading to a long-term risk of CVD and poor prognosis (30). Mechanisms confirmed by experiments mainly include promoting macrophage foam-cell formation by interfering reverse cholesterol transport (3), bile acid metabolism disorder (31), proinflammatory cytokine production and vascular inflammation (30), and platelet hyperactivity to thrombosis (32). Along with the thorough research on gut microbiota and CVDs, TMAO has been studied deeply, providing a promising targeted therapy for CVDs in future. Wang et al. (33) found that 3,3-dimethyl-1-butanol (DMB), a structural choline analog, could non-lethally inhibit TMA formation, reduce TMAO levels, and further decrease diet-induced atherosclerosis. Unfortunately, our study failed to find a significant correlation between TMAO and CHD risk in the whole population, unlike most previous studies. Patients’ dietary habits, recent history of antibiotic use, or renal function might be taken into consideration. However, this motivated us to further explore the roles of sex and age differences on the relationship between TMAO and CHD. Through our research, a sex–age difference in the association between plasma TMAO concentration and CHD was reported. The results suggest we take sex and age differences into consideration when evaluating TMAO–CHD association in the future. It has important clinical implications. If further verified, our study may provide crucial data for clinical monitoring and treatment. It will guide us to evaluate the clinical significance of TMAO based on the sex and age of patients, which conforms to the concepts of precision medicine and personalized treatment.

The study inevitably has several limitations. First, it is a cross-sectional observational study, so causality may not be demonstrated clearly. Second, as a single-center study, with more rigorous inclusion criteria for patients with CHD, the result may not be generalizable to other populations. It will be more convincing to use two methods to assess severity of CHD. Further studies in other hospitals and by other means are warranted. In addition, plasma TMAO is a dynamic gut product, influenced by dietary intake, gut microbiota, and host health (9). We did not have detailed food or medication (e.g., antibiotics, prebiotics) intake information and this might partly confound the result. Finally, TMA, rather than TMAO, was also indicated as being involved in the CVD pathology mechanism by reducing cardiomyocytes viability in older rats (34). It will be beneficial to know the relationship between TMA and CHD risk according to sex and age. But TMA is unstable, and thus our preserved blood samples could not be used for measurement. Future studies involving sensitive and stale-detecting methodologies for metabolites are required.

## Conclusions

Our study reveals a significantly positive association between plasma TMAO and CHD risk and severity in Chinese men, but not in women. Age affects the relationship between plasma TMAO and CHD risk in men. Possible mechanisms for these differences might involve dietary habits, gut microbiota background, metabolic characteristics, and organ function. Further longitudinal investigations are required to confirm and explain our findings. Our study will be helpful to accurately understand the mechanisms of TMAO in the progression of CVD, and promote precise intervention against CVD in different populations.

## Data Availability

The original contributions presented in the study are included in the article/Supplementary Material, further inquiries can be directed to the corresponding author.
