# Supplementary material for: Association between plasma trimethylamine N-oxide and coronary heart disease: new insights on sex and age differences
Source: Front Cardiovasc Med. 2024 Oct 7;11:1397023. doi: 10.3389/fcvm.2024.1397023 (PMC11491342; doi:10.3389/fcvm.2024.1397023)
Supplement: Supplementary file 1 [file Datasheet1.pdf]

Supplementary Table 1. Characteristics of cases and control participants

Characteristic*	Overall	Controls	Cases	*P* value
N	858	429	429	
Female (%)	456 (53.1)	228 (53.1)	228 (53.1)	1
Age, years	63.5 ± 10.4	63.1 ± 10.3	63.9 ± 10.5	0.241
BMI, kg/m^2	26.1 ± 3.7	26.2 ± 3.7	25.9 ± 3.7	0.298
SBP, mmHg	132.9 ± 16.5	132.1 ± 16.2	133.6 ± 16.9	0.161
DBP, mmHg	74.9 ± 11.1	75.5 ± 11.4	74.2 ± 10.7	0.098
Smoke status (%)				0.005
Never	490 (59.5)	268 (64.6)	222 (54.3)	
Ever	153 (18.6)	62 (14.9)	91 (22.2)	
Current	181 (22.0)	85 (20.5)	96 (23.5)	
Drinking status (%)				1
Never	587 (70.7)	297 (70.7)	290 (70.7)	
Ever	83 (10.0)	42 (10.0)	41 (10.0)	
Current	160 (19.3)	81 (19.3)	79 (19.3)	
**Comorbidities, N (%)**				
Hypertension	600 (69.9)	280 (65.3)	320 (74.6)	0.003
Diabetes	361 (42.1)	139 (32.4)	222 (51.7)	<0.001
Hyperlipidemia	669 (78.0)	317 (73.9)	352 (82.1)	0.004
Family history of CHD	294 (36.9)	140 (35.6)	154 (38.2)	0.449
**Medication use, N (%)**				
Antihypertensive	476 (55.5)	226 (52.7)	250 (58.3)	0.099
Glucose-lowering	263 (30.7)	97 (22.6)	166 (38.7)	< 0.001
Lipid-lowering	411 (47.9)	171 (39.9)	240 (55.9)	< 0.001
**Laboratory biomarkers**				
Hemoglobin, g/L	135.4 ± 15.0	136.2 ± 14.4	134.7 ± 15.5	0.135
Glucose, mmol/L	7.0 ± 3.0	6.5 ± 2.8	7.5 ± 3.4	<0.001
LDL-C, mmol/L	2.33 ± 0.81	2.39 ± 0.82	2.28 ± 0.81	0.017
HDL-C, mmol/L	1.06 ± 0.25	1.09 ± 0.25	1.04 ± 0.25	<0.001
Hcy, μmol/L	13.4 (10.3-17.3)	13.5 (10.0-17.7)	13.4 (10.5-16.9)	0.859
Creatinine, μmol/L	75.2 (64.7-87.5)	74.2 (63.8-84.9)	76.4 (66.0-89.7)	0.016
D-Dimer, mg/L	0.08 (0.05-0.14)	0.08 (0.05-0.14)	0.09 (0.05-0.15)	0.207
TMAO, μg/mL	0.11 (0.06, 0.18)	0.10 (0.06-0.17)	0.11 (0.07-0.18)	0.277

Abbreviations: CHD = coronary heart disease; BMI = body mass index; SBP = systolic blood pressure; DBP = diastolic blood pressure; LDL-C = low density lipoprotein cholesterol; HDL-C = high density lipoprotein cholesterol; Hcy = homocysteine; TMAO = trimethylamine N-oxide;

*Data are presented as number (%) or mean ± Stand deviation or median (interquartile range) depending on the distribution.

Supplementary Table 2. Characteristics of cases and control participants in different sex subgroups

Characteristic*	Male			Female		
	Controls	Cases	*p*-value	Controls	Cases	*P* value
N	201	201		228	228	
Age, years	59.9 ± 11.3	60.3 ± 11.5	0.753	65.8 ± 8.3	67.1 ± 8.3	0.110
BMI, kg/m^2	26.4 ± 3.5	25.9 ± 3.3	0.097	26.0 ± 3.9	26.0 ± 3.9	0.988
SBP, mmHg	132.2 ± 15.9	132.2 ± 16.3	0.970	132.0 ± 16.5	134.9 ± 17.3	0.066
DBP, mmHg	78.3 ± 12.3	76.7 ± 10.5	0.153	73.0 ± 10.0	72.1 ± 10.4	0.334
Smoke status (%)			0.033			<0.001
Never	57 (29.2)	37 (19.2)		211 (95.9)	185 (85.6)	
Ever	61 (31.3)	80 (41.5)		1 (0.5)	11 (5.1)	
Current	77 (39.5)	76 (39.4)		8 (3.6)	20 (9.3)	
Drinking status (%)			0.940			0.675
Never	81 (41.3)	82 (42.9)		216 (96.4)	208 (95.0)	
Ever	39 (19.9)	38 (19.9)		3 (1.3)	3 (1.4)	
Current	76 (38.8)	71 (37.2)		5 (2.2)	8 (3.7)	
**Comorbidities, N (%)**						
Hypertension	125 (62.2)	142 (70.6)	0.073	155 (68.0)	178 (78.1)	0.015
Diabetes	64 (31.8)	85 (42.3)	0.030	75 (32.9)	137 (60.1)	<0.001
Hyperlipidemia	150 (74.6)	165 (82.1)	0.069	167 (73.2)	187 (82.0)	0.025
Family history of CHD	60 (32.6)	75 (38.9)	0.206	80 (38.3)	79 (37.6)	0.890
**Medication use, N (%)**						
Antihypertensive	98 (48.8)	108 (53.7)	0.318	128 (56.1)	142 (62.3)	0.182
Glucose-lowering	41 (20.4)	63 (31.3)	0.012	56 (24.6)	103 (45.2)	<0.001
Lipid-lowering	70 (34.8)	108 (53.7)	<0.001	101 (44.3)	132 (57.9)	0.004
**Laboratory biomarkers**						
Hemoglobin, g/L	143.9 ± 13.2	143.7 ± 13.2	0.859	129.3 ± 11.6	126.7 ± 12.8	0.022
Glucose, mmol/L	6.4 ± 2.2	6.8 ± 2.7	0.129	6.5 ± 2.7	8.1 ± 3.9	<0.001
HDL-C, mmol/L	0.99 ± 0.21	0.97 ± 0.22	0.321	1.17 ± 0.25	1.09 ± 0.25	<0.001
LDL-C, mmol/L	2.32 ± 0.78	2.22 ± 0.77	0.243	2.46 ± 0.84	2.33 ± 0.83	0.112
Hcy, μmol/L	14.3 (11.2-19.0)	14.8 (11.4-19.0)	0.995	12.7 (9.4-16.2)	12.3 (9.9-15.0)	0.987
Creatinine, μmol/L	83.0 (74.7-92.7)	85.9 (77.2-95.7)	0.076	65.0 (59.3-74.9)	68.1 (60.5-77.3)	0.048
D-Dimer, mg/L	0.07 (0.05-0.12)	0.07 (0.05-0.14)	0.565	0.09 (0.06-0.15)	0.10 (0.06-0.16)	0.233
TMAO, μg/mL	0.10 (0.06-0.17)	0.10 (0.06-0.17)	0.778	0.10 (0.06-0.19)	0.12 (0.07-0.20)	0.220

Abbreviations: CHD = coronary heart disease; BMI = body mass index; SBP = systolic blood pressure; DBP = diastolic blood pressure; LDL-C = low density lipoprotein

cholesterol; HDL-C = high density lipoprotein cholesterol; Hcy = homocysteine; TMAO = trimethylamine N-oxide;
*Data are presented as number (%) or mean ± standard deviation or median (interquartile range) depending on the distribution.

Supplementary Table 3. Association between plasma TMAO and CHD risk in patients stratified by sex and age

Tertiles of TMAO, ug/mL	Cases/controls	Crude Model		Adjusted Model		*P* for interaction
		OR (95%CI)	*P* value	OR (95%CI)	*P* value	
**Male**						
**< 65 years**	122/133					0.028
T1 (≤ 0.072)	40/42	Ref.		Ref.		
T2 (0.072-0.133)	45/43	1.10 (0.60, 2.01)	0.759	1.11 (0.56, 2.19)	0.767	
T3 (> 0.133)	37/48	0.81 (0.44, 1.49)	0.496	0.71 (0.36, 1.42)	0.336	
P for trend		0.492		0.323		
**≥ 65 years**	79/68					
T1 (≤ 0.075)	20/28	Ref.		Ref.		
T2 (0.075-0.146)	29/21	1.93 (0.87, 4.32)	0.108	1.89 (0.71, 5.01)	0.202	
T3 (> 0.146)	30/19	2.21 (0.98, 4.98)	0.056	2.70 (1.03, 7.09)	0.044	
P for trend		0.056		0.044		
**Female**						
**< 65 years**	91/112					0.426
T1 (≤ 0.066)	23/43	Ref.		Ref.		
T2 (0.066-0.129)	32/36	1.66 (0.83, 3.33)	0.152	1.52 (0.71, 3.26)	0.279	
T3 (> 0.129)	36/33	2.04 (1.02, 4.08)	0.044	1.53 (0.68, 3.44)	0.301	
P for trend		0.045		0.293		
**≥ 65 years**	137/116					
T1 (≤ 0.092)	48/34	Ref.		Ref.		
T2 (0.092-0.178)	43/43	0.71 (0.38, 1.30)	0.268	0.82 (0.39, 1.69)	0.586	
T3 (> 0.178)	46/39	0.84 (0.45, 1.54)	0.565	1.00 (0.47, 2.13)	1	
P for trend		0.574		0.966		

Abbreviations: CHD = coronary heart disease; TMAO = trimethylamine N-oxide; BMI = body mass index; Hcy = homocysteine.

Adjusted model was adjusted for BMI, smoking status, drinking status, hypertension diagnosis, diabetes diagnosis, hyperlipidemia diagnosis, family history of CHD, creatinine and Hcy.

Supplementary Table 4. Association between plasma TMAO and higher Gensini score in CHD patients stratified by sex

TMAO, ug/mL	N	Crude Model		Adjusted Model	
		OR (95% CI)	*P* value	OR (95% CI)	*P* value
**Overall**					
Continuous†	429	1.05 (0.90, 1.21)	0.560	1.04 (0.86, 1.25)	0.686
Tertiles					
T1 (≤ 0.080)	143	Ref.		Ref.	
T2 (0.080 - 0.152)	143	0.89 (0.56, 1.42)	0.635	0.87 (0.50, 1.50)	0.621
T3 (> 0.152)	144	0.97 (0.61, 1.55)	0.903	0.97 (0.56, 1.69)	0.918
P for trend		0.904		0.919	
**Male**					
Continuous†	201	1.11 (0.89, 1.40)	0.350	1.00 (0.77, 1.31)	0.972
Tertiles					
T1 (≤ 0.075)	67	Ref.		Ref.	
T2 (0.075 - 0.139)	67	1.09 (0.55, 2.17)	0.802	1.01 (0.45, 2.26)	0.986
T3 (> 0.139)	67	0.99 (0.50, 1.96)	0.983	0.74 (0.33, 1.66)	0.471
P for trend		0.981		0.461	
**Female**					
Continuous†	228	1.03 (0.84, 1.26)	0.807	0.98 (0.74, 1.31)	0.916
Tertiles					
T1 (≤ 0.083)	76	Ref.		Ref.	
T2 (0.083 - 0.159)	76	0.69 (0.36, 1.31)	0.253	0.52 (0.22, 1.20)	0.125
T3 (> 0.159)	76	0.95 (0.50, 1.79)	0.871	0.77 (0.32, 1.85)	0.556
P for trend		0.871		0.568	

†TMAO value was log_2-transformed.

Abbreviations: CHD = coronary heart disease; TMAO = trimethylamine N-oxide; BMI = body mass index; Hcy = homocysteine.

Adjusted model was adjusted for sex (only for overall population), age, BMI, smoking status, drinking status, hypertension diagnosis, diabetes diagnosis, hyperlipidemia diagnosis, family history of CHD, creatinine and Hcy.

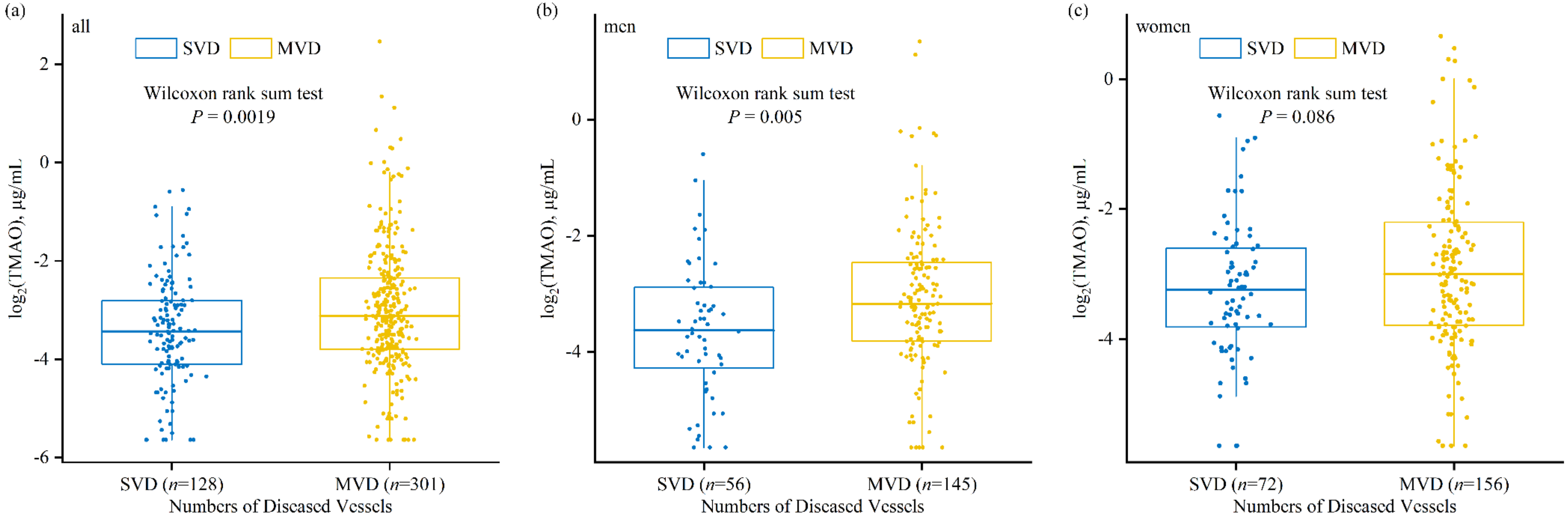

Supplementary Figure 1. Plasma $\log_2$(TMAO) levels between single-vessel disease (SVD) group and multiple-vessel disease (MVD) group in CHD patients.

(a) Overall population; (b) Men; (c) Women.

Abbreviations: CHD = coronary heart disease; TMAO = trimethylamine N-oxide; MVD = multiple-vessel disease; SVD = single-vessel disease.
